# A cross-sectional study of sub-clinical *Plasmodium falciparum* infection in HIV-1 infected and uninfected populations in Mozambique, South-Eastern Africa

**DOI:** 10.1186/1475-2875-11-252

**Published:** 2012-08-01

**Authors:** Emilia V Noormahomed, Marika Orlov, Virgilio do Rosario, Brett W Petersen, Carly Guthrie, Roberto Badaro, Robert T Schooley

**Affiliations:** 1 Department of Microbiology, Universidade Eduardo Mondlane, Maputo, Mozambique; 2 Department of Medicine, University of California, San Diego, San Diego, CA, USA; 3 Instituto Higiene e Medicina Tropical, Universidade Nova de Lisboa, Lisbon, Portugal; 4 Department of Medicine, Universidade Federal da Bahia, Salvador, Brazil; 5 Current address: Division of Infectious Diseases, University of California, San Diego, Mail Stop 0711, 9500 Gilman Dr, La Jolla, CA, 92093, USA

**Keywords:** Malaria, *P. falciparum*, HIV-1

## Abstract

**Background:**

*Plasmodium falciparum* and HIV-1 infection cause substantial morbidity and mortality in sub-Saharan Africa. Increasing evidence suggests these two pathogens interact negatively when infecting the same individual.

**Methods:**

A cross-sectional study among HIV-1 infected and uninfected populations was recruited in Mocuba and Maputo, Mozambique to determine the prevalence of sub-clinical malarial parasitaemia using light microscopy and a nested PCR assay.

**Results:**

The prevalence of sub-clinical *P. falciparum* parasitaemia was low in Maputo, whether determined by microscopy (0.4%) or PCR (1.9%), but substantially higher in Mocuba (7.6 and 14.7%, respectively). Nested PCR detected nearly 70% more cases of sub-clinical parasitaemia than microscopy, but differences occur by locality. HIV-1 infected persons were more likely to be sub-clinically parasitaemic than HIV-1 uninfected individuals recruited from the same geographic areas. Trimethoprim-sulphamethoxazole use did not substantially reduce sub-clinical parasitaemia.

**Conclusions:**

Dried blood spots are a convenient and sensitive technique for detecting sub-clinical infection with *P. falciparum* by nested PCR*.* Prevalence of *P. falciparum* is substantially lower in Maputo where malaria control programmes have been more active than in the rural town of Mocuba. In Mocuba, among those presenting for HIV-1 counseling and testing, the prevalence of *P. falciparum* is substantially higher in those who test positive for HIV-1 than those without HIV-1 infection. The clinical implications of sub-clinical *P. falciparum* infection among HIV-1 infected persons warrant additional study**.**

## Background

Malaria and HIV each cause substantial morbidity and mortality in sub-Saharan Africa, where they extensively overlap geographically. It is estimated that in 2009 approximately 2.5 million deaths were attributable to malaria and/or HIV-1 infection [1,2]. Although it was initially thought that the two epidemics were independent, more recent studies demonstrate that these two pathogens negatively impact each other from several perspectives [3-5]. During clinically apparent bouts of *Plasmodium falciparum* infection, plasma HIV RNA levels increase [6]. CD4^+^ T lymphocytes decline temporarily during clinical malaria episodes in both HIV-infected and uninfected patients [7]. Repeated malaria infections are associated with a more rapid decline in CD4^+^ T lymphocyte counts over time in HIV-1 infected individuals. These findings suggest that symptomatic *P. falciparum* infections may accelerate progression of HIV disease [7]. Conversely, recent studies indicate that persons with HIV-1 infection, especially those with more advanced disease, are at greater risk for clinical malaria [8,9]. In addition, bouts of clinical malaria in HIV-infected patients, are more likely to be associated with increased morbidity and mortality and higher relapse rates following therapy [10].

Over the past several years, Mozambique’s national malaria control programme has increased its efforts to reduce morbidity and mortality from malaria. These efforts have included intra-domiciliary spraying, the distribution of impregnated bed nets (especially for pregnant women and children below five years of age), and intermittent chemoprophylaxis in pregnant women. These measures have contributed to reductions in malaria-related morbidity and mortality, especially in urban areas of the country. Clinical cases of malaria reported to Mozambique’s Ministério da Saúde (Ministry of Health) declined from 6,155,082 in 2007 to 4,091,614 by 2009. Also, deaths attributed to malaria decreased from 3,889 to 2,311 over the same time interval [11,12]. Despite these reductions, malaria remains a major cause of morbidity and mortality in Mozambique, especially among women and children, and still accounts for 44% of outpatient visits, 57% of pediatric admissions and 26% of all hospital admissions [13].

Mozambique also ranks among the top tier of countries in HIV-1 with an estimated adult prevalence rate of 14% (in those 15-49 years of age)[14]. Mozambique, therefore, serves as an excellent venue in which to further delineate interactions between these two pathogens. The prevalence of sub-clinical malaria (which is defined as having microbiological evidence of infection without any clinical symptoms) was studied in two locations in Mozambique. This study was conducted in an effort to better understand the prevalence of sub-clinical *P. falciparum* parasitaemia in two representative locations within Mozambique by blood smear and by polymerase chain reaction (PCR) technology. In addition, the study examined whether sub-clinical parasitaemia was affected by HIV-1 infection and/or trimethoprim-sulphamethoxazole (TS) prophylaxis.

## Methods

### Study design

This was a prospective cross-sectional study designed to determine the prevalence of sub-clinical malaria in HIV-1 infected and uninfected control volunteers residing in similar locations. In addition, relationships among malarial parasitaemia, HIV status and anti-malarial intervention efforts were examined.

### Study sites and populations sampled

Study participants were recruited from two hospitals in Maputo (the Polana Caniço Hospital and the Maputo Military Hospital) and from the Mocuba District Hospital, which served as a representative rural location in Zambeze province.

### Enrollment procedures and sample collection

Volunteers who were at least 18 years of age and seeking care or voluntary testing for HIV-1 were recruited for this study. Prior to enrollment, a nurse or HIV counselor explained the study to participants and invited their participation. Volunteers who agreed to participate and provided written informed consent were enrolled into the study. The National Bioethics Committee of Mozambique and the Human Research Protections Program of the University of California, San Diego, approved the study. Demographic and clinical data including gender, age, CD4 cell count, HIV-1 treatment history and the use of selected concomitant medications (including trimethoprim-sulphamethoxazole [TS] were recorded. Patients were also asked if they had received therapy for malaria and, if so, how recently and what drug was administered. Capillary blood was obtained by finger stick from those seeking HIV testing. One spot of blood was put onto a slide, stained with Giemsa and examined by optical microscopy [15]. Approximately fifty uL of blood was also placed on Whatman # 2 filter paper and allowed to air dry. The blood spot was then fixed in methanol and air dried prior to storage at 4° C until shipment to San Diego, where they were stored at -20° C until processing for PCR.

### Detection of *P. falciparum* DNA by PCR

Half of each blood spot was used to extract DNA using a saponin/chelex extraction. Briefly, the DBS was placed in 0.5% saponin in PBS over night at 4° C. The next day the saponin solution was aspirated and replaced with 1 mL of PBS and placed at 4° C for 15-30 minutes. The DBS was then placed into pre-heated 5% Chelex water solution, vortexed and incubated for 10 minutes at 100° C. Vortexing was repeated five minutes through the incubation, and at the end of the incubation. Tweezers were used for all transfers of the DBS between tubes and were flamed between each transfer. The DBS/Chelex solution was then centrifuged at 10,000 g for 2 min and the supernatant was moved to a new tube and was either used for PCR or stored at -20° C.

The PCR for *P. falciparum* detects a 700 bp portion of the Merozoite Specific Protein 2 (*msp*2) gene. Reactions were performed using 2μL of supernatant from the DBS DNA extraction in a 50μL PCR reaction (2.5 U Taq polymerase [Invitrogen], 3 mM MgCl2, 500nM dNTPs [Fermentas], and 50 pmol of each primer [IDT]). In the first round of the nested PCR, the gene was amplified using primers *msp*2_1F (5’-GAAGGTAATTAAAACATTGTC-3’) and *msp*2_1R (5’-GAGGGATGTTGCTGCTCCACAG-3’). For the PCR, denaturation was performed at 94° C for 5 minutes, followed by 30 cycles of denaturation at 94° C for 30 seconds, annealing at 55° C for 2 minutes, and elongation at 70° C for 2 minutes, with a final extension at 72° C for 7 minutes. 1μL of the outer PCR product was used as the template for the inner PCR reaction using primers MSP2_2F (5’-GAGTATAAGGAGAAGTATG-3’) and MSP2_2R (5’-CTAGAACCATGCATATGTCC-3’), using the same reaction times and temperatures. The PCR products from the inner PCR were run on a 1% agarose gel at 175 V for 30 minutes and stained with ethidium bromide for visualization.

### Detection of malarial parasites by light microscopy

Two independent microscopists read the smears for malaria parasites. A third microscopist who was blinded to the original results examined slides with discordant results and the final results of the parasitaemia were the average of the two similar counts. Results were expressed as the number of visible parasites/μL blood.

### HIV testing

HIV testing was performed in parallel with two rapid HIV antibody detection kits (Determine [Abbott Laboratories] and Unigold [Trinity Biotech]).

### Data analysis

Data were analysed using EPI INFO (version 3.4.3-CDC-Atlanta). Continuous variables were compared using Student’s t tests for variables not normally distributed. Proportions were compared using the *X*^2^ test or the Fisher’s exact test. A 2-tailed P value of ≤ 0.05 was judged to be significant. Univariate and stratified analyses of data on age, sex, parasite density, and HIV serostatus, use of TS, antiretroviral therapy regimen (ART) and fever, CD4 cell count were conducted to identify potential confounders and effect modifiers of the associations between the two pathogens. Statistical measure of inter-rater reliability was calculated using Fleiss' kappa index formula [16,17].

## Results

### Study population demographics

**Figure 1:**
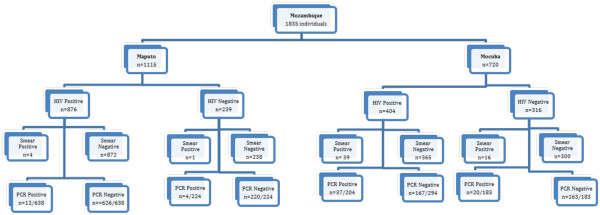
- Testing algorithm for study participants.

The algorithm shown in Figure 1 presents the overall screening for HIV and malaria testing of the entire study population. Overall 1,835 persons were recruited (Maputo, n = 1,115, and Mocuba n = 720) of whom 1,280 (69.8%) were HIV infected and 555 (30.2%) were HIV seronegative. The mean age of the study population was 32.6 years (range, 18 – 79) for the HIV infected cohort and 30.7 years (range, 18 – 66) for the uninfected cohort. The HIV infected cohort was 64% female and 36% male.

Table 1 summarizes the prevalence of *P. falciparum* by microscopy and the prevalence of *P. falciparum* DNA by nested PCR by location. A greater proportion of those recruited in Maputo (78.6%) than Mocuba (56.1%) were HIV-1 infected.

**Table 1:** **Frequency of positivity for *Plasmodium falciparum *by direct smear examination or of *P. falciparum *DNA by nested PCR by location **

			**Maputo n = 1115**		**Mocuba n = 720**		**Total (both sites) n = 1835**	
			**n**	**(%)**	**n**	**(%)**		**(%)**
**HIV Positive**			876	(68.0)	404	(32.0)	1280	(100)
	Smear		876		404		1280	(100)
		Positive	4	(0.5)	39	(9.6)	43	(3.3)
		Negative	872	(99.5)	365	(90.4)	1237	(96.7)
	PCR		638		204		842	(100)
		Positive	12	(1.9)	37	(18.1)	49	(5.8)
		Negative	626	(98.1)	167	(81.9)	793	(94.2)
**HIV Negative**			239	(43.0)	316	(57)	555	(100)
	Smear		239		316		555	(100)
		Positive	1	(0.4)	16	(5.1)	17	(3.1)
		Negative	238	(99.6)	300	(94.9)	538	(96.9)
	PCR		224		183		407	(100.0)
		Positive	4	(1.8)	20	(10.9)	24	(5.9)
		Negative	220	(98.2)	163	(89.1)	383	(94.1)
**Total**								
		Smear	1115	(61)	720	(39)	1835	(100)
		Positive	5	(0.5)	55	(7.6)	60	(3.2)
		Negative	1110	(99.6)	665	(92.4)	1775	(96.8)
		PCR	862		387		1249	(100)
		Positive	16	(1.8)	53	(13.7)	69	(6.0)
		Negative	846	(96.5)	334	(86.3)	1198	(94.0)

Blood smears from all 1,835 study participants were examined by light microscopy for malarial parasites and the results are presented in Table 1. PCR for *P. falciparum* DNA was performed on a subset of 1249 (68.1%) of the study population. The overall prevalence of malaria by microscopy was higher in Mocuba than in Maputo (5.1% vs. 0.4%, p <0.001). Likewise *P.m falciparum* DNA was detected more frequently in Mocuba (53 of 387 study participants, 14.7%) than in Maputo (16 of 862 study participants (1.9%) (p = 0.001).

### Comparison of parasitaemia rates in the HIV-1 infected and uninfected populations

In Mocuba, blood smear positivity for *P. falciparum* was significantly higher among HIV infected individuals compared to HIV uninfected individuals [39/404 (9.9%) vs. 16/316 (5.1%), p <0.02]. Although the same trend toward a higher prevalence of malaria in the HIV infected population than in the uninfected population was noted when assessed by PCR [(37/204, 18.2%) and (20/183, 10.9%)], the difference did not reach statistical significance (p < 0.1). In Maputo only 0.5% of the 1115 study participants were smear positive for *P. falciparum* and only 16 of the 862 (1.8%) had detectable *P. falciparum* DNA in plasma. In those with blood smears that were positive for malarial parasites, there was a trend toward a higher mean parasitaemia level in HIV-1 infected study participants (11786 ± 28267 parasites/μl) compared to those who were not infected with HIV-1 (2425 ± 4701 parasites/μl) (p = 0.08).

### Impact of TS and ART use on parasitaemia

National HIV treatment guidelines at the time of the study recommended that HIV-1 infected persons with <250 CD4 cells/mm^3^ be prescribed chronic TS prophylaxis. During the period of the study, however, TS was out of stock in Maputo and thus few Maputo participants received the drug. In Mocuba information about TS prophylaxis was available for 397 of the 404 participants whose blood smears were examined for parasites and from 201 of the 204 examined by PCR (Table 2). Parasitaemia rates were similar by smear 10.7 vs. 8.4% for those on TS prophylaxis compared to those not on TS respectively. Similarly, *P. falciparum* DNA was detected at a similar rate in the two groups (14.3 vs. 17.9%) respectively. Information about antiretroviral therapy status and malarial positivity was available for 335 participants from Mocuba. Parasitaemia rates were similar between those who reported receiving antiretroviral drugs (9/87, 10.3%) and those not reporting use of antiretroviral drugs (30/248, 12%).

**Table 2:** Sensitivity, specificity and efficiency of PCR to detect malaria in the study population

**Test**		**Smear**		**Total**	**Sen.**	**Spec.**	**Eff.**
		**Positive**	**Negative**				
PCR	Positive	38	35	73	0.90	0.97	0.97
	Negative	4	1,172	1,176			
Total		42	1,207	1,249			

Data represent the number of positive and negative individuals results for samples tested by both methods. Sen = Sensitivity, Spec = specifcity and Eff = efficiency. Data are the proportion of true positive and true negative (using the peripheral blood smear as the gold standard test).

### Comparison of the sensitivity and Kappa index of light microscopy and PCR

Using visual inspection of the peripheral blood smear as the gold standard reference for malaria parasite detection, the sensitivity and specificity of PCR is presented in Table 3. There is a substantial agreement between the smear and PCR for detection of malaria k = 0.646 [0.543-0.748]. Indeed, the efficiency of PCR for a positive or negative result is 97%. Overall, PCR detected malarial parasitaemia in 73 of 1249 (5.8%) study participants compared to 60 of 1835 (3.3%) participants evaluated by light microscopy (*X*^2^ = 10.9, p < 0.01). This difference in sensitivity was driven by the HIV-1 infected population where the rate of parasitaemia was 49/842 (5.8%) as assessed by PCR but only 43/1,280(3.4%) when assessed by microscopy. By contrast, the rates of parasitaemia were comparable (~3%) in the HIV-1 uninfected population whether assessed by microscopy or PCR. *Plasmodium falciparum* DNA was detected by PCR in 38 of the 42 (90.4%) samples that were positive by microscopy. In contrast, parasites were detected by microscopy in only 38 of the 73 (52%) of those that had *P. falciparum* DNA detected by PCR analysis.

**Table 3:** Effect of trimethoprim-sulphamethoxazole prophylaxis on malarial positivity by direct smear examination and PCR among participants enrolled in Mocuba

	**On TS**	**Not on TS**	**TS Status Unknown**	**Total**
Smear Positive	3 (10.7%)	31 (8.4%)	5 (71.4%)	39
Smear Negative	25 (89.3%)	338 (91.6%)	2 (28.6%)	365
Total	28	369	7	404
PCR Positive	4 (14.3%)	31 (17.9%)	2 (66.7%)	37
PCR Negative	24 (85.7%)	142 (82.1%)	1 (33%)	167
Total	28	173	3	204

## Discussion

In this study, sub-clinical malaria was more prevalent in HIV-1 infected individuals than in HIV-1 seronegative populations when assayed by either smear or nested DNA PCR. Sub-clinical parasitaemia was less frequent in Maputo where malaria eradication efforts have been more intensive over the past several years in comparison with rural areas of the country. The significantly higher rate of sub-clinical malaria in asymptomatic individuals from Mocuba and the use of TS prophylaxis in HIV-1 infected persons with lower CD4 cell counts provided an opportunity to evaluate the impact of TS prophylaxis on the prevalence of sub-clinical malaria by both light microscopy and PCR. Although the practice of prescribing TS prophylaxis primarily to those with lower CD4 cell counts was a potential confounding factor, our data indicate that TS prophylaxis does not eliminate sub-clinical parasitaemia whether assessed by microscopy or PCR. Among those who were smear positive for malaria, more than twofold higher parasite density levels were observed in the HIV-1 infected population than among those who were HIV-1 seronegative, in contrast to what has been previously reported [18]. Although both populations were recruited from people seeking care from HIV-1 testing and treatment sites in the same clinical facilities, caution in interpreting the differences in prevalence and parasitaemia levels between the HIV-1 infected and HIV-1 seronegative populations is warranted since other differences in the two populations might be unrecognized.

It is also possible that the observed differences in prevalence and levels of parasitaemia between the HIV-1 infected and uninfected populations might actually underestimate the effect of HIV-1 infection on malarial parasitaemia rates since HIV-1 infected individuals since those under care for HIV might be more likely to be in contact with the health care system and have more access to bed nets and other preventive measures. Furthermore, since syndromic treatment of fever with empiric anti-malarial therapy is not uncommon, persons with fever from HIV-1 or its complications could be more likely to receive empiric intermittent anti-malarial therapy for other disease states which might further lower the prevalence of sub-clinical malaria in this group. An additional limitation of the study was that HIV RNA levels are not routinely monitored in Mozambique and, thus, viral load data were not available.

TS prophylaxis in HIV patients reduces morbidity and mortality from malaria as well as from *Pneumocystis jirovecii*[19,20]. In view of the low prevalence of malarial parasitaemia and of clinical malaria in Maputo, TS would seem to be less critical from the perspective of reducing morbidity and mortality from malaria than in places like Mocuba, where the risk of malaria remains higher. The potential impact of TS on other HIV-1 related opportunistic infections, such as *Pneumocystis jirovecii* pneumonia would, nonetheless, continue to be important. It is well demonstrated that HIV infection predisposes to more frequent and more severe episodes of malaria [3,5,9,10]. Since this was a cross-sectional rather than a longitudinal study, time-dependent malaria-related morbidity and mortality rates were beyond the scope of this study. Nonetheless, in Mocuba where *P. falciparum* elimination efforts have been less successful than in Maputo, it was found that the HIV cohort had 1.7 times more sub-clinical malaria than the HIV uninfected cohort as defined by PCR. Others have demonstrated that overt clinical bouts of malaria are associated with increased levels of HIV-1 RNA and of CD4 cell decline [6,21]. The implications of sub-clinical malarial parasitaemia for HIV-1 disease progression have not yet been delineated, but it would seem that such studies are warranted [22,23]. Finally, although the impact of sub-clinical malarial parasitaemia on the risk of clinical malaria in either the HIV-1 infected or the HIV-1 seronegative population or on the progression of HIV-1 in the co-infected population has not been fully delineated, the sensitivity of nested PCR of dried blood spots for detecting carriage of *P. falciparum* might make this an attractive approach to monitor the success or failure of malaria control efforts in areas, such as Mocuba, as more concerted efforts to eliminate malaria are undertaken.

## Competing interests

Dr. Schooley is a member of the Scientific Advisory Board of Gilead Sciences.

## Authors’ contributions

EVN and RTS designed, obtained research funding and were responsible for the overall conduct of the study. RB and VR contributed to data analysis and preparation of the manuscript. BP, CG and MO contributed to study design and analysis and were responsible for sample collection and conducted the PCR assays. All authors read and approved the final manuscript.
